# A Standardized Traditional Chinese Medicine Preparation Named Yejuhua Capsule Ameliorates Lipopolysaccharide-Induced Acute Lung Injury in Mice via Downregulating Toll-Like Receptor 4/Nuclear Factor-*κ*B

**DOI:** 10.1155/2015/264612

**Published:** 2015-03-23

**Authors:** Chu-Wen Li, Zhi-Wei Chen, Xiao-Li Wu, Zhao-Xiao Ning, Zu-Qing Su, Yu-Cui Li, Zi-Ren Su, Xiao-Ping Lai

**Affiliations:** ^1^School of Chinese Materia Medica, Guangzhou University of Chinese Medicine, Guangzhou Higher Education Mega Center, No. 232, Waihuandong Road, Guangzhou 510006, China; ^2^Institute of Chinese Medical Sciences, University of Macau, Macau; ^3^Dongguan Mathematical Engineering Academy of Chinese Medicine, Guangzhou University of Chinese Medicine, Songshan Lake High-Tech Industrial Development Zone, Dongguan, Guangdong 523808, China; ^4^Faculty of Health Science, University of Macau, Macau; ^5^The First Affiliated Hospital, Guangzhou University of Traditional Chinese Medicine, Guangzhou 510405, China

## Abstract

A standardized traditional Chinese medicine preparation named Yejuhua capsule (YJH) has been clinically used in treatments of various acute respiratory system diseases with high efficacy and low toxicity. In this study, we were aiming to evaluate potential effects and to elucidate underlying mechanisms of YJH against lipopolysaccharide- (LPS-) induced acute lung injury (ALI) in mice. Moreover, the chemical analysis and chromatographic fingerprint study were performed for quality evaluation and control of this drug. ALI was induced by intratracheal instillation of LPS (5 mg/kg) into the lung in mice and dexamethasone (5 mg/kg, p.o.) was used as a positive control drug. Results demonstrated that pretreatments with YJH (85, 170, and 340 mg/kg, p.o.) effectively abated LPS-induced histopathologic changes, attenuated the vascular permeability enhancement and edema, inhibited inflammatory cells migrations and protein leakages, suppressed the ability of myeloperoxidase, declined proinflammatory cytokines productions, and downregulated activations of nuclear factor-*κ*B (NF-*κ*B) and expressions of toll-like receptor 4 (TLR4). This study demonstrated that YJH exerted potential protective effects against LPS-induced ALI in mice and supported that YJH was a potential therapeutic drug for ALI in clinic. And its mechanisms were at least partially associated with downregulations of TLR4/NF-*κ*B pathways.

## 1. Introduction

Acute lung injury (ALI) or acute respiratory distress syndrome (ARDS) is a severe illness complicated with systemic inflammatory responses in the airspaces and lung parenchyma, which involves alveolar-capillary membrane damage, vascular permeability increase, neutrophils recruitment, pulmonary edema, and respiratory failure [1–3]. Clinically, ALI is a life-threatening problem and always leads to multiple organ dysfunctions syndrome (MODS), with significant incidences and mortalities in critically ill patients [2, 3]. Although several candidate therapy strategies have been applied for ALI [1, 4, 5], there is still no effective therapy strategy but relatively noteworthy mortality rates [3, 6, 7]. Therefore, to develop novel effective preventions and therapies is urgently required.

The pathologies of ALI have been generally defined as uncontrolled and excess productions of inflammatory mediators such as cytokines, chemokines, adhesion molecules, and bioactive lipid products [1–3]. And lipopolysaccharide (LPS), major constituent of outer membranes of Gram-negative bacteria, is a pivotal risk factor and prominent stimulus for inflammatory mediators releasing [8]. LPS would induce neutrophil infiltrations, trigger acute inflammatory responses, and generate early lung pathological changes, which would lead to the morbidity and development of ALI [9]. Nowadays, with clearly clinical relations to the process of ALI, intratracheal instillation of LPS to experimental animals has been demonstrated to be a well-suited and reproducible model for preliminarily pharmacological researches of novel drugs or other therapeutic agents [9, 10]. Once entering into the host, LPS would trigger the innate immune systems by activating toll-like receptors superfamily (TLRs) [5, 11]. Among TLRs, TLR4 is a major LPS receptor and mediates the most inflammation responses to LPS by activating nuclear factor (NF)-*κ*B protein, via myeloid differentiation factor 88- (MyD88-) dependent and MyD88-independent pathway [5, 11, 12]. Then, the activated NF-*κ*B induces the productions of proinflammatory cytokines such as tumor necrosis factor-*α* (TNF-*α*), interleukin-1*β* (IL-1*β*), and IL-6 [11–13], resulting in recruitments of intravascular neutrophils into the alveolar space and lung parenchyma [14, 15]. Hence, drugs focusing on downregulating the TLR4 signaling pathways inflammatory responses and/or inhibiting other related oxidative stress would provide potential therapeutic effects for ALI [5, 11].

Nowadays, increasing attentions have being focused on the ethnological medicinal plants as they are affordable for a wide range of inflammatory diseases with less toxicities and adverse effect [16, 17]. And in China, Chrysanthemi Indici Flos, Patchouli Oil (PO, the essential oil extracted from Herba Pogostemonis), and Zedoary Turmeric Oil (ZTO, the essential oil extracted from Curcume Rhizoma) are three very important herbal materials that are extensively used to treat respiratory disorders among the common traditional Chinese medicine (TCM) [18–20].

Chrysanthemi Indici Flos is a traditional herbal remedy extensively applied in China to treat various inflammation-related disorders for thousands of years [18–20]. Currently, the supercritical-carbon dioxide fluid extraction of Chrysanthemi Indici Flos (SFEC) has been widely used as a fine material in many TCM preparations with high anti-inflammatory efficacy and low toxicity [18, 21]. And there were reports indicating that some bioactive compounds (e.g., chlorogenic acid, galuteolin, linarin, luteolin, and apigenin) in SFEC had potential protective effects against ALI [22–25].

PO is a famous herbal medicine and has been widely used in TCM as it offers several notably pharmaceutical features for many diseases treatments [20]. It has been reported to have antioxidant, antinociceptive, antiallergy, antibacterial, and antirespiratory virus actions [26–29]. The chemistry profiles of PO indicated that patchoulol alcohol and pogostone are the two most components in it [27, 28]. Previous studied also demonstrated that patchouli alcohol could significantly prevent LPS-induced ALI in mice [30–33].

Similarly, ZTO, the essential oil extracted from a time-honored medicine Curcume Rhizoma, is now an important medical material in China [20, 34, 35]. Researches revealed that ZTO could ameliorate inflammatory response via blocking infiltrations and edemas [34, 35]. Additionally, this oil had the antivirus effects against herpes simplex virus, influenza virus, adenovirus, and so forth [34, 35]. Moreover, several bioactive terpenoids and alkenes from ZTO, especially, germacrone, had been reported to possessed potential anti-ALI features in animal models [36].

Currently, a standardized traditional Chinese herbal medicine compound preparation derived from some famous and confirmed recipes, named Yejuhua capsule (YJH), consisting of SFEC, PO, and ZTO, has been used in treatments of several acute respiratory system diseases, including cold, cough, acute bronchitis, acute laryngitis, and acute pharyngitis, with high efficacy and low toxicity [21]. Previous studies also demonstrated that YJH possessed remarkable anti-influenzal, anti-inflammatory, analgesia, and antipyretic effects as well as the regulation of immune systems [21]. Pretreatments of YJH could significantly improve the survival rate in virus-infected mice. And its effects on acute inflammation have also been proved to be associated with regulations of several proinflammatory cytokines, such as TNF-*α*, IL-1*β*, and IL-6.

Although YJH has been shown anti-inflammatory benefits in acute respiratory system diseases, its potential effects to protect against LPS-induced ALI still remained unclear. Also, in this present study, we aimed to evaluate the effects of YJH against LPS-induced ALI in mice. And for potential mechanisms elucidation, the expressions of TLR4 as well as the phosphorylations of NF-*κ*B p65 and inhibitory (I) *κ*B *α* were also evaluated. Meanwhile, for the quality evaluation and control, chemical analysis and chromatographic fingerprint study of YJH were also conducted.

## 2. Materials and Methods

### 2.1. Chemicals and Reagents and Plant Materials

Authentic standard compounds including chlorogenic acid, galuteolin, linarin, luteolin, apigenin, patchoulol alcohol, pogostone, germacrone, curdione, and furanodiene were purchased from National Institutes for Food and Drug Control (Beijing, China). Lipopolysaccharide (LPS, from* Escherichia coli* 0111:B4) and dexamethasone (Dex) were purchased from Sigma Co., Ltd. (St. Louis, USA) and XianJu Pharmaceutical Co., Ltd. (Zhejiang, China), respectively. Phosphate buffered saline (PBS), sodium dodecyl sulphate polyacrylamide gel (SDS-PAGE), skimmed milk, Tween-20, and Tween-80 were purchased from Thermo-Fisher Sci. Co., Ltd. (MA, USA). Hexadecyltrimethylammonium bromide (HTAB) and* o*-dianisidine were purchased from TCI Co., Ltd. (Tokyo, Japan). All other chemicals were of the reagent grade.

SFEC (Lot. RD20120507) was prepared and provided by the Institute of New Drug Research and Development, Guangzhou University of Chinese Medicine (GZUCM), according to the previous research [18]. Briefly, Chrysanthemi Indici Flos was smashed and then extracted using the 532 supercritical fluid extract apparatus (Light Industry Institute of Guangzhou, Guangdong, China) with the total extract time at 4 h, the flow rate of CO_2_ at 20 L/h, the pressure at 25 MPa, the temperature at 45°C, and the modifier of 95.0% ethanol (10.0% of the sample weight). PO and ZTO were purchased from Nanhai Zhongnan Co., Ltd. (Guangdong, China, Lot. 120601), and Dazhou Natural Plant Pharmaceutical Co., Ltd. (Sichuan, China, Lot. 20120709A), respectively. Both PO and ZTO were produced by using the method of steam distillation.

### 2.2. Preparation of YJH

YJH was prepared according to the previous research [21]. In brief, SFEC, PO, and ZTO were mixed at the ratio of 3 : 3 : 1 (w/w). After that, the mixture was made into soft capsules with appropriate amounts of medical gelatin and medical glycerin. The weight of the content in each capsule is about 280 mg. And samples of single constituent (SFEC, PO, and ZTO) were prepared according the procedure above, respectively. For the quality evaluation study, 10 batches of YJH (Lot. number YJH 1301-YJH 1310) were prepared and analyzed.

In this paper, for pharmacological tests, the contents of YJH were carefully taken out from soft capsules and dissolved in 0.5% Tween-80 solution. To perform the chromatographic fingerprints, 100 mg of the contents (samples from YJH and single constituent) was dissolved in pure ethanol and metered to a total volume of 10 mL.

### 2.3. GC-MS, UPLC-PAD, and Fingerprint Analysis of YJH

The YJH capsule contains various kinds of bioactive constituents, including flavonoids, terpenoids, phenolic compounds, and alkenes [18, 28, 31, 32]; therefore, a method of Gas Chromatography-Mass Spectrometer (GC-MS) analysis combining with Ultraperformance Liquid Chromatography-Photodiode Array Detector (UPLC-PAD) analysis was established for chemical and fingerprinting analysis. In our study, three relevant bioactive compounds, patchoulol alcohol in PO, germacrone, curdione in ZTO, were selected for GC-MS analysis and nine relevant bioactive compounds, chlorogenic acid, galuteolin, linarin, luteolin and apigenin in SFEC, pogostone in PO and germacrone, curdione, and furanodiene in ZTO, were selected for UPLC-PAD analysis. At the same time, fingerprint profiles of GC-MS and UPLC-PAD were also successfully established for the quality assessments of YJH capsule.

#### 2.3.1. Apparatus and GC-MS Analysis

GC-MS analysis was performed on an Agilent 6890-5975 GC-MS system (Agilent, Palo Alto, USA). The chromatographic separations were carried out via a 5% phenyl methyl siloxane HP-5MS capillary column (30 m × 0.25 mm inner diameter, 0.25 *μ*m film). The oven temperature program was optimized and conducted as follows: initially at 60°C, followed by raise to 100°C (10°C/min, held for 1 min), next, programmed to 110°C (1°C/min, held for 1 min), and after this elevated to 150°C (3°C/min, held for 1 min) and finally to 260°C (10°C/min, held for 5 min). Split injection (1.0 *μ*L) was conducted with a split ratio of 60 : 1 and helium was used as carrier gas with 1.0 mL/min flow rate. The spectrometer was selected in electron-impact (EI) mode, and the ionization energy, scan ranges, and scan rate were set in 70 eV, 40 to 400 amu, and 0.34 s per scan, respectively. The temperatures of inlet and ionization source were 230°C and 250°C, respectively. Identifications of the main compounds (patchoulol alcohol, germacrone, and curdione) were based on comparisons of retention times and mass spectra data with those of authentic samples. The content of each compound in sample was quantified based on the peak area integrated by the analysis programs.

#### 2.3.2. Apparatus and UPLC-PAD Analysis

HPLC-PAD analysis was carried out on a Shimadzu LC-20A HPLC system consisting of a SPD-M20A PDA detector, a LC-20AT pump, a SIL-20AC automatic sampler, a CTO-20A thermostatic column compartment, and a Shimadzu LC-20A software (Shimadzu, Kyoto, Japan). The separation was performed on a Phenomenex Synergi Hydro-RP 80A C18 column (2.0 × 150 mm, 4 *μ*m, Phenomenex Inc., CA, USA) with a flow rate of 0.3 mL/min, temperature at 30°C, and injection volume of 5 *μ*L. The mobile phase consisted of acetonitrile (A) and 0.05% aqueous phosphoric acid (B) was used with the gradient mode (0–3 min: 15% A→18% A; 3–20 min: 18% A→24% A; 20–30 min: 24% A→28% A; 30–40 min: 28% A→30% A; 40–65 min: 30% A→38% A; 65–70 min: 38% A→46% A; 70–80 min: 46% A→48% A; 80–100 min: 48% A→52% A; 100–103 min: 52% A→62% A; 103–120 min: 62% A→95% A; 120–130 min: 95% A→15% A). The absorption spectra of compounds were recorded from 190 to 800 nm. Identifications of the main compounds (chlorogenic acid, galuteolin, linarin, luteolin, apigenin, pogostone, germacrone, curdione, and furanodiene) were based on comparisons of the retention time and the ultraviolet (UV) absorption (190 to 800 nm) data with those of authentic samples. The contents of these compounds were quantitative analyses with peak areas under the standard curves at an optimized UV-wavelength, 242 nm.

#### 2.3.3. Evaluation of GC-MS and UPLC-PAD Fingerprint Analysis

Fingerprint analysis was performed by the Similarity Evaluation System for Chromatographic Fingerprint of Traditional Chinese Medicine (version 2004A). The correlative coefficients and the similarity indexes of chromatograms (GC-MS and UPLC-PAD) from 10 batches of YJH with the common chromatogram were calculated and compared.

### 2.4. Experimental Animal

Male Kunming [5] mice (20–25 g) were purchased from Medical Laboratory Animal Center of Guangdong Province (Certificate number SCXK2008-0002, Guangdong Province, China). Animals were kept on 12-hour light/12-hour dark cycle under regular temperature (22 ± 2°C) and humidity (50 ± 10%) with standard diets and clean water* ad libitum*. All animals were sacrificed by lethal sodium pentobarbital injection. All experiments were conducted according to the National Institutes of Health Guide for the Care and Use of Laboratory Animals and approved by the Institutional Animal Care and Use Committee of GZUCM.

### 2.5. Experimental Design of ALI

#### 2.5.1. Protocol of Mouse Equivalent Doses

Currently, the body surface area- (BSA-) based dose calculation is the most appropriate and basic method for dose conversions in animal experiment [37]. Therefore, in this study the conversions of the human equivalent dose (HED) into mouse equivalent dose [38] were calculated according to the previous study by using the following formula for dose translation based on BSA [37]:

(1) $$\begin{array}{c} \text{HED}\left(\text{mg}/\text{kg}\right)=\text{MED}\left(\text{mg}/\text{kg}\right)\times \left(\frac{\text{Mouse}\;\;\text{Km}}{\text{Human}\;\;\text{Km}}\right), \end{array}$$

where Mouse Km = 3 and Human (adult) Km = 37.

The proposed dosage of Yejuhua capsule is 3 capsules (equivalent to 840 mg YJH sample) for an adult (60 kg of weight) per day in common case, which is equivalent to the dose of 14 mg/kg. And in this study, for pharmacological tests, three human doses of YJH used in clinic with curative effect, including 7, 14, and 28 mg/kg, were selected and used. The MEDs of these HEDs were translated to 85 mg/kg, 170 mg/kg, and 340 mg/kg, per day, according to the previous formula [37].

#### 2.5.2. Model of LPS-Induced ALI

Mice were randomly divided into 6 groups (*n* = 30): sham group, LPS group, YJH (85, 170, and 340 mg/kg) groups, and Dex group (5 mg/kg). YJH groups and Dex group were given YJH (85, 170, and 340 mg/kg, p.o.) and Dex (5 mg/kg, p.o.) once per day for 7 consecutive days, respectively. During this period, sham group and LPS group were given equal volumes of 0.5% Tween-80. One hour after the last administration, mice were anesthetized via intraperitoneally injecting pentobarbital sodium (30 mg/kg). After that, mice from LPS group, Dex group, and YJH groups were given a single intratracheal instillation of 5 mg/kg LPS (2.5 mg/mL, freshly diluted with PBS; 20 *μ*L/10 g body weight), while mice of sham group were given an equal volume of PBS. In this model, all animals survived for 24 hours after the intratracheal instillation of LPS at the dose of 5 mg/kg, which was optimized and repeatable, based on our preliminary experiments (data were not provided).

#### 2.5.3. Protocol of Specimen Collection

24 hours after LPS instillation, 30 mice of each group were randomly divided into 3 parts, 10 mice per part. Part 1 was used for the bronchoalveolar lavage fluid (BALF) preparation and the lung wet/dry weight (W/D) ratio measurement. In brief, mouse was anaesthetized and surgically operated to expose the trachea. Then the right main bronchus was clamped and the left lung was lavaged for three times with a total volume of 1.5 mL of Hanks-Balanced-Salt solution using a venous indwelling needle. The BALF recovery rate was more than 90%. Then the mouse was sacrificed, and right lung was harvested for the W/D ratio measurement. Part 2 was used for the lung tissue preparation and the histopathologic evaluation. In brief, after the mouse was sacrificed, the left lung was taken, placed in appropriate amount of precold PBS immediately, and homogenized using a TissueLyser II high-throughput tissue homogenization system (Qiagen Co., Ltd., Hilden, Germany). Then the homogenate was centrifuged at 4°C. 100 *μ*L of the supernatant was used for protein measurement and the rest were immediately collected and stored at −80°C for further analysis of MPO and proinflammatory cytokines (TNF-*α*, IL-1*β*, and IL-6). At the same time, the right lung was harvested for the histopathologic examination. Part 3 was left for western blot assay of TLR4, NF-*κ*B p65, and I*κ*B *α*. In brief, mouse was sacrificed, and the lung tissues were harvested, frozen, and stored in liquid nitrogen immediately.

#### 2.5.4. Measurement of Survival Rate

To assess mortality rate, 120 mice were randomly divided into 5 groups (*n* = 24): sham group, LPS group, and YJH (85, 170, and 340 mg/kg) groups. YJH groups were given YJH (85, 170, and 340 mg/kg, p.o.), while sham group and LPS group were given Tween-80, for 7 consecutive days. One hour after the last administration, all animals were anesthetized. And mice from LPS group and YJH groups were given a single intratracheal instillation of 20 mg/kg LPS (10 mg/mL, dilution with PBS; 20 *μ*L/10 g body weight), while mice from the sham group were given an equal volume of PBS. After operation, mice of all groups were monitored and the time when anyone animal died was recorded every 6 hours up to 120 hours. Then the mortality rate of each group within 120 hours was calculated and compared using the Kaplan Meier methods.

### 2.6. Measurement of Lung W/D Ratio

The lung W/D ratio was measured according to the previous study. In brief, the excised right lung was blotted dry and weighed to obtain the “wet” weight and afterwards kept in an oven at 80°C for 48 hours to obtain the “dry” weight. Then the W/D ratio was calculated by the “wet” weight to the “dry” weight.

### 2.7. Measurement of BALF Protein Contents and BALF Cell Counts

The BALF was centrifuged at 800 ×g for 10 min at 4°C, and the supernatant was collected for measurement of protein content. Measurements of protein contents of BALF were performed using a commercial BCA kit (Beyotime Institute of Biotechnology, Shanghai, China) and expressed as mg/mL BALF. Then sediment cells were resuspended in precold PBS and stained by a Wright-Giemsa kit (Nanjing Jiancheng Bioengineering Institute, Nanjing, China) for cytospin preparations. Counts of the total cells, neutrophils, and macrophages were then double-blindly performed via hemocytometer.

### 2.8. MPO Assay

The assay of MPO activity was performed via the HTAB method. Briefly, the samples were mixed with KPO_4_ buffer (50 mM, pH 6.0) with HTAB (0.5%). After reacting and incubating at 37°C for 15 min, the enzyme was assayed by the activity in a H_2_O_2_/*o*-dianisidine buffer at 460 nm with a Multiskan GO microplate spectrophotometer (Thermo-Fisher Sci., Waltham, USA). Results were expressed as U/mg protein.

### 2.9. Histopathologic Examination

Biopsies of right lungs were collected, fixed in 4% paraformaldehyde solution, dehydrated, embedded with paraffin, and sectioned into 4 *μ*m. Tissue sections were stained with hematoxylin and eosin kit (H&E, Beyotime Institute of Biotechnology), examined, and photographed using TE2000-S Inverted Microscopes (Nikon Co., Ltd., Tokyo, Japan).

### 2.10. ELISA Assay for TNF-*α*, IL-1*β*, and IL-6

TNF-*α*, IL-1*β*, and IL-6 were measured using commercially available ELISA kits (*e* Bioscience Co., Ltd., CA, USA). In brief, diluted standards or samples were added to 96-well plates precoated with affinity purified polyclonal-antibodies specific for mouse TNF-*α*, IL-1*β*, and IL-6, respectively. Then wells were added with enzyme-linked polyclonal antibodies and incubated at 37°C for 60 min, followed by final washes for 5 times. The intensities detected at 450 nm were measured after addition of substrate solutions for 15 min. Levels of TNF-*α*, IL-1*β*, and IL-6 were calculated according to standard curves.

### 2.11. Western Blot Assay for TLR4, I*κ*B *α*, and NF-*κ*B p65

Extractions of proteins from the lung tissues were performed with T-PER tissues proteins extractions reagent kits (Beyotime Institute of Biotechnology). Extractions of nuclear and cytoplasmic proteins from the lungs were performed with nuclear and cytoplasmic proteins extractions reagent kits (Beyotime Institute of Biotechnology). Protein contents were measured using BCA protein assay kits and equal amounts of protein were added in per well on 10% SDS PAGE. Then, proteins were separated and transferred into PVDF membranes by an Electrophoresis System (Bio-Rad Co., Ltd., Hercules, USA). The resulting membranes were blocked with Tris-buffered-saline containing 0.05% Tween-20 (TBS-T), supplemented with 5% skimmed milk at room temperature for 2 hours and followed by TBS-T washings. Then membranes were incubated with related specific primary antibodies anti-NF-*κ*B p65 antibody, anti-I*κ*B *α* antibody (Cell Signaling Technology Co., Ltd., MA, USA), and anti-TLR4 antibody (Santa Cruz Co., Ltd., TX, USA) at 4°C overnight, respectively, followed by washes with TBS-T and incubation with the peroxidase-conjugated secondary antibody at room temperature for 1 hour. The detections of labeling proteins were performed with enhanced-chemiluminescence western-blotting detections kits. And the relative protein levels were normalized to *β*-actin (Santa Cruz Co., Ltd.) protein as the internal standard.

### 2.12. Statistical Analysis

Data were presented as the mean ± SEM and statistical analyses were performed with Systat SigmaPlot software (version 12.00 for windows). Parametric data were analyzed by one-way ANOVA, followed by Tukey-Kramer test, and nonparametric data were analyzed by Kruskal-Wallis test, followed by Dunn's test. The mortality studies were analyzed by the Kaplan-Meier method. And *P* < 0.05 was considered to be statistically significant.

## 3. Results

### 3.1. Results of GC-MS, UPLC-PAD, and Fingerprint Analysis

**Figure 1:**
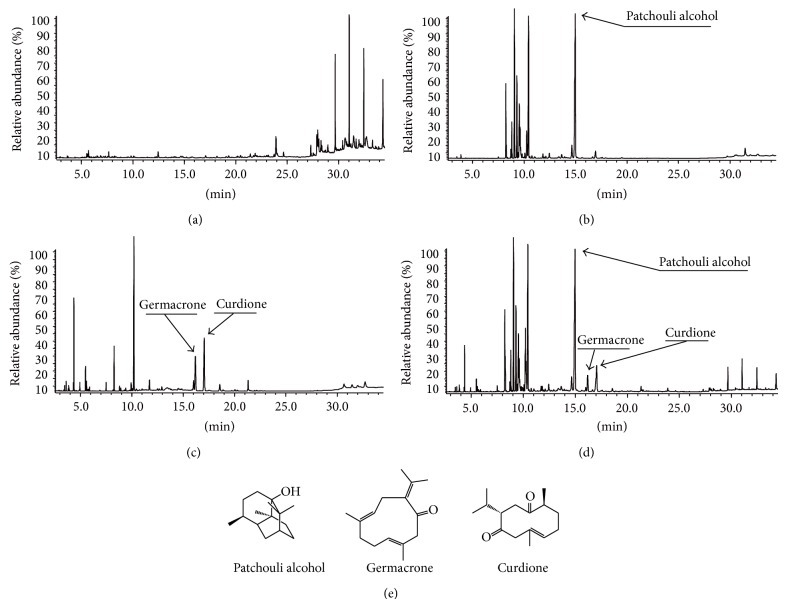
The GC-MS chromatograms of the Yejuhua capsule and its three ingredients by GC-MS analysis. GC-MS chromatograms of the Yejuhua capsule and its three ingredients. SFEC: supercritical-carbon dioxide fluid extraction of Chrysanthemi Indici Flos (a), PO: Patchouli Oil (b), ZTO: Zedoary Turmeric Oil (c), and YJH: Yejuhua capsule (d). The structures of authentic standard compounds used in the GC-MS analysis (e).

GC-MS chromatograms of SFEC, PO, ZTO, and YJH were achieved via the proposed chromatography conditions and shown in Figure 1. Three compounds including patchouli alcohol, germacrone, and curdione were identified in YJH samples, and the average contents of these compounds in 10 batches samples are 108.42 mg/g, 21.36 mg/g, and 16.05 mg/g, respectively.

**Figure 2:**
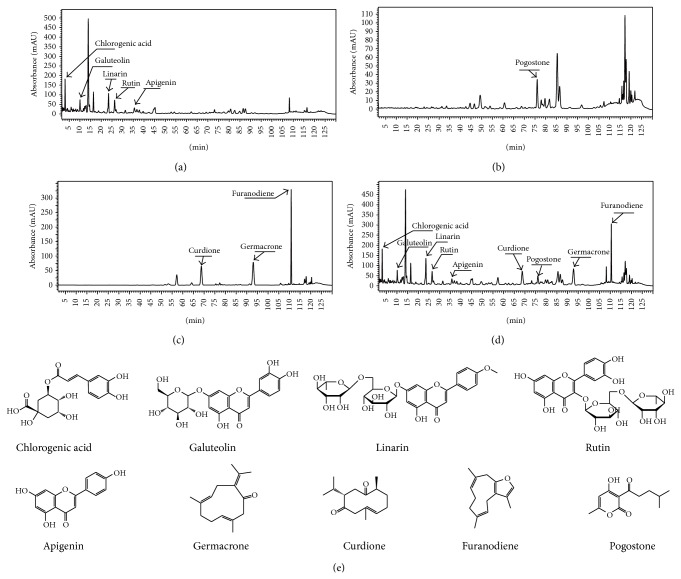
The UPLC-PAD chromatograms of the Yejuhua capsule and its three ingredients by UPLC-PAD analysis. The UPLC-PAD chromatograms of the Yejuhua capsule and its three ingredients. SFEC: supercritical-carbon dioxide fluid extraction of Chrysanthemi Indici Flos (a), PO: Patchouli Oil (b), ZTO: Zedoary Turmeric Oil (c), and YJH: Yejuhua capsule (d). The structures of authentic standard compounds used in the UPLC-PAD analysis (e).

UPLC analysis of YJH and other samples were achieved using the proposed conditions. Based on the number of chromatographic peaks detected and the response values of these chromatographic peaks, a UV-wavelength of 242 nm was optimized as the detection wavelength for the further content determination and UPLC fingerprint analysis. The HPLC chromatograms of SFEC, PO, ZTO, and YJH samples at the detection wavelength of 242 nm were shown in Figure 2. In this section, a total of nine compounds including chlorogenic acid, galuteolin, linarin, luteolin, apigenin, pogostone, germacrone, curdione, and furanodiene were identified in the HPLC-PAD analysis profiles at the wavelengths from 190 to 800 nm. And the average contents of these compounds in 10 batches of YJH samples are 9.23 mg/g, 9.87 mg/g, 22.65 mg/g, 11.25 mg/g, 4.83 mg/g, 21.36 mg/g, 16.05 mg, and 27.14 mg/g, respectively.

**Figure 3:**
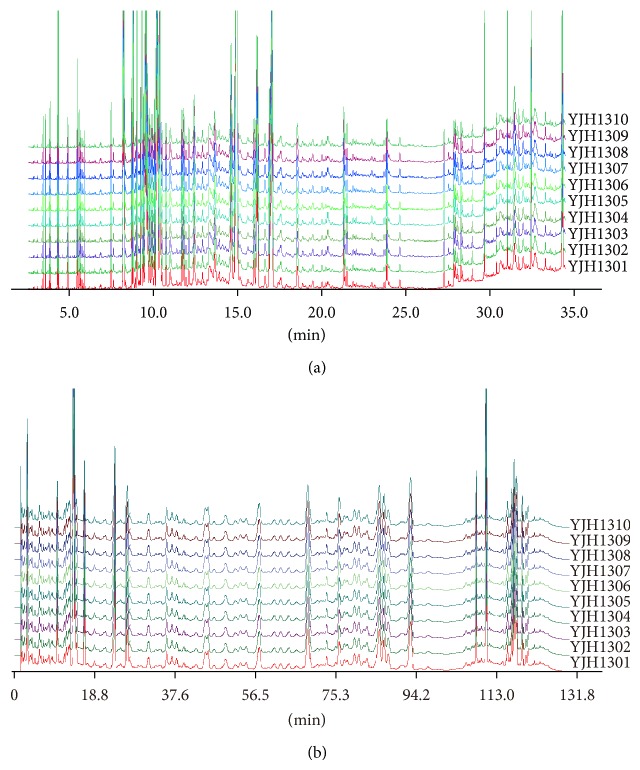
The GC-MS and UPLC-PAD fingerprinting chromatograms of 10 batches of Yejuhua capsule. The GC-MS fingerprinting chromatograms of 10 batches of Yejuhua capsule (a) and the UPLC-PAD fingerprinting chromatograms of 10 batches of Yejuhua capsule (b). YJH131-YJH1310: batch of Yejuhua capsule sample from number 1 to number 10.

For fingerprint analysis, Similarity Evaluation System was applied for the fingerprint analysis and similarity evaluation. The similarity indexes of GC-MS fingerprint analysis (shown in Figure 3(a)) and UPLC fingerprint analysis (shown in Figure 3(b)) for YJH samples (a total of 10 batches) varied from 0.960 to 0.982 and from 0.973 to 0.994, respectively, which indicated a good correlation and stable quality of YJH samples.

### 3.2. Effects of YJH on Mortality Rate

**Figure 4:**
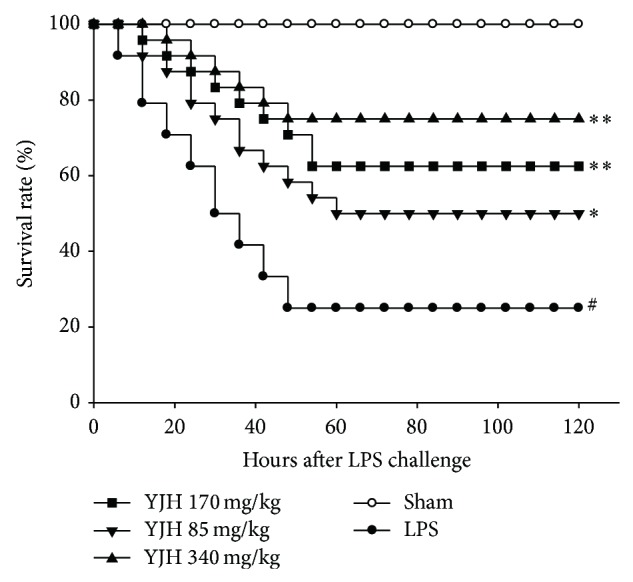
Effects of YJH on survival rate. Data was represented as the mean ± SEM (*n* = 24). ^#^ *P* < 0.01 compared to the sham group, ^*^ *P* < 0.05 and ^**^ *P* < 0.01 compared to the LPS group.

As shown in Figure 4, compared to the shame group, LPS challenge markedly declined the survival rate of the LPS group, maintaining at 25.0% within 120 hours (*P* < 0.01). Conversely, survival rates of YJH groups (85, 170, and 340 mg/kg) significantly increased to 75.0%, 62.5%, and 50.0%, respectively, in a dose-dependent manner (for all, *P* < 0.05 versus the LPS group). Results indicated that YJH possessed potential prevention of mortality in ALI mice induced by LPS.

### 3.3. Effects of YJH on Lung W/D Ratio

**Figure 5:**
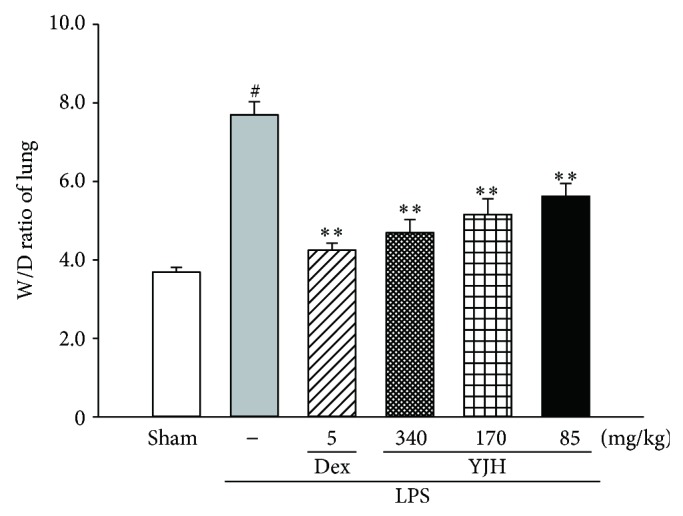
Effects of YJH on lung W/D ratio. Data was represented as the mean ± SEM (*n* = 10). ^#^ *P* < 0.01 compared to the sham group, ^*^ *P* < 0.05 and ^**^ *P* < 0.01 compared to the LPS group.

The W/D ratio of ALI mice was evaluated to assess the severity of pulmonary edema. And as shown in Figure 5, when compared with the sham group, there was a significant increase (approximate 2-fold) in the lung W/D ratio of the LPS group (*P* < 0.01). However, with the treatments of YJH (85, 170, and 340 mg/kg), the levels of lung W/D ratio were dose-dependently suppressed, as compared to the LPS group (for all, *P* < 0.01). Data showed that the lung W/D ratio was significantly suppressed by pretreatment with YJH.

### 3.4. Effects of YJH on BALF Protein Content

**Figure 6:**
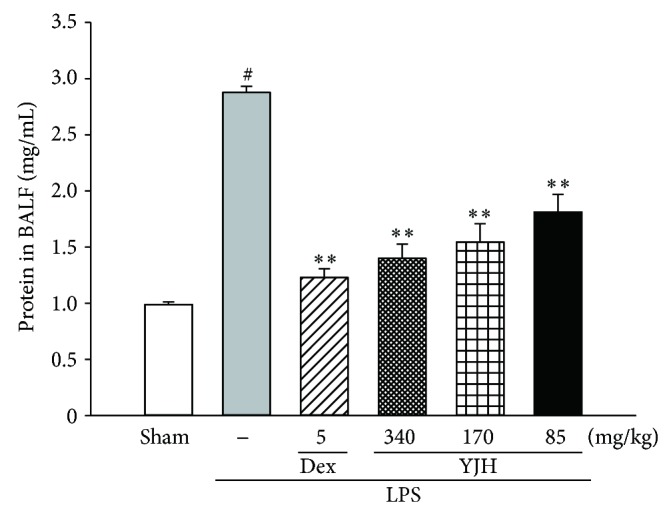
Effects of YJH on BALF protein content. Data was represented as the mean ± SEM (*n* = 10). ^#^ *P* < 0.01 compared to the sham group, ^*^ *P* < 0.05 and ^**^ *P* < 0.01 compared to the LPS group.

The vascular permeability of the lung in mice was measured by the protein content of BALF. As shown in Figure 6, LPS led to a significant increase in the BALF protein level of the LPS group, as compared to the sham group (*P* < 0.01). On the contrary, the protein contents of YJH groups (85, 170, and 340 mg/kg) were markedly suppressed in a dose-dependent manner, when compared with the LPS group (for all, *P* < 0.01).

### 3.5. Effects of YJH on Cell Counts of BALF

**Figure 7:**
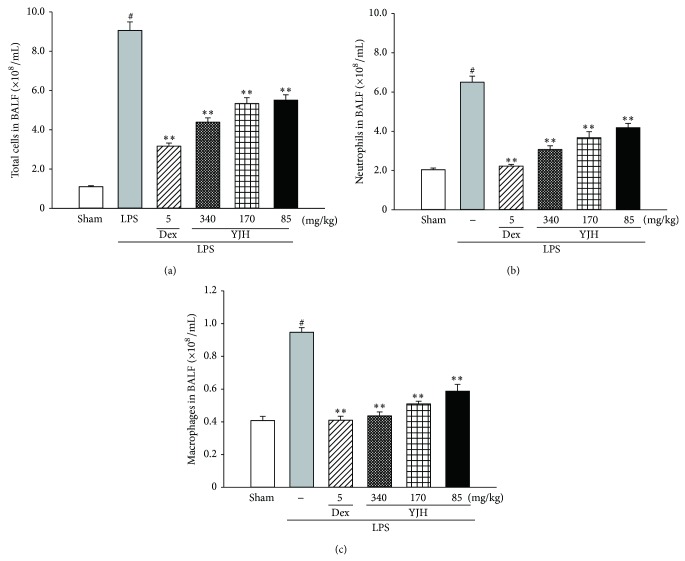
Effects of YJH on total cells, neutrophils, and macrophages in the BALF. Total cells (a), neutrophils (b), and macrophages (c) in the BALF. Data was represented as the mean ± SEM (*n* = 10). ^#^ *P* < 0.01 compared to the sham group, ^*^ *P* < 0.05 and ^**^ *P* < 0.01 compared to the LPS group.

As shown in Figure 7, the BALF cell counts of LPS group showed significant increases in the total cells (Figure 7(a)), neutrophils (Figure 7(b)), and macrophages (Figure 7(c)) (*P* < 0.01 versus the sham group). However, pretreatments with YJH (85, 170, and 340 mg/kg) and Dex (5 mg/kg) markedly decreased all relevant cell counts in YJH groups and Dex group, respectively (for all, *P* < 0.01 versus the LPS group).

### 3.6. Effects of YJH on MPO Activity

**Figure 8:**
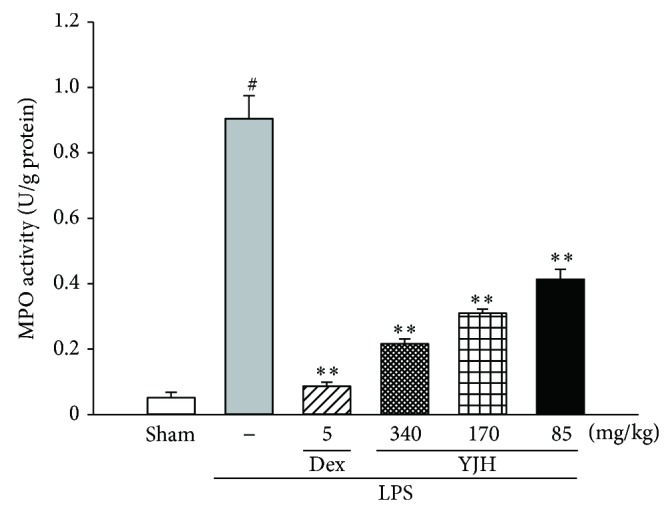
Effects of YJH on MPO activity. Data was represented as the mean ± SEM (*n* = 10). ^#^ *P* < 0.01 compared to the sham group, ^*^ *P* < 0.05 and ^**^ *P* < 0.01 compared to the LPS group.

MPO activity, served as a functional index indicating the infiltration of neutrophils, which represented the levels of MPO-derived oxidants generations and lung tissue damages. As a result of the LPS challenge, the lung MPO activity in LPS group was significantly elevated, about 9-fold of the sham group (*P* < 0.01, versus the sham group), which was shown in Figure 8. However, the activities of MPO of the YJH and Dex groups were significantly inhibited by the treatments of YJH (85, 170, and 340 mg/kg) and Dex (5 mg/kg), respectively (for all, *P* < 0.05 versus the LPS group).

### 3.7. Effects of YJH on Histopathological Examination

**Figure 9:**
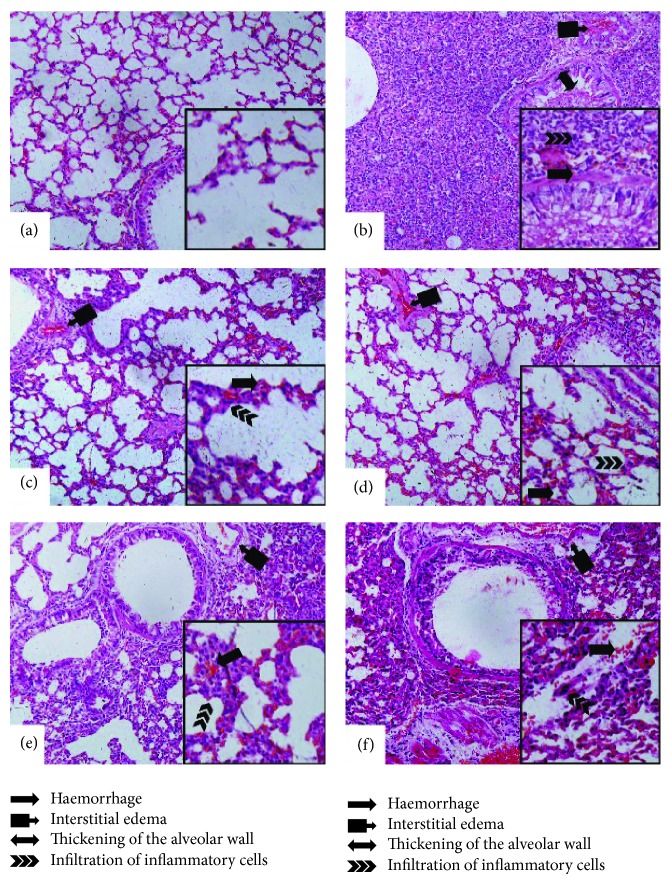
Effects of YJH on histopathological examination. Sham (a), LPS (b), LPS + 5 mg/kg Dex (c), LPS + 120 mg/kg YJH (d), LPS + 800 mg/kg YJH (e), and LPS + 40 mg/kg YJH (f) (200x and 400x).

Histopathological analyses were performed to investigate the effects of YJH on physiological parameters. As expected, the sham group displayed normal structures and no histopathologic changes in lung tissues (Figure 9(a)). On the other hand, with the challenge of LPS, the pulmonary function of LPS group was obviously impaired, with various histopathological changes including haemorrhage, interstitial edema, thickening of the alveolar wall, and infiltration of inflammatory cells into the lung parenchyma and alveolar spaces (Figure 9(b)). As for experimental groups and positive group, histopathological changes were obviously abated by pretreatments of Dex (5 mg/kg, Figure 9(c)) and YJH (85 mg/kg, Figure 9(d); 170 mg/kg, Figure 9(e); and 340 mg/kg, Figure 9(f)), respectively, when compared to the LPS group. Results demonstrate that YJH attenuated the severity of lung injuries and improved the condition of lungs tissues, in LPS-induced ALI mice, dose-dependently.

### 3.8. Effects of YJH on TNF-*α*, IL-1*β*, and IL-6 Levels

As compared to the sham group, the levels of TNF-*α*, IL-1*β*, and IL-6 in LPS group were remarkably raised (for all, *P* < 0.05), shown in Table 1. Administration with YJH (170 and 340 mg/kg) had inhibitory effects on the levels of TNF-*α* (for both, *P* < 0.05, versus the LPS group), although that at dose of 85 mg/kg did not reduce this level statistically. In addition, YJH (85, 170, and 340 mg/kg) downregulated the protein levels of IL-1*β* and IL-6, as compared to the LPS group (for all, *P* < 0.05).

### 3.9. Effects of YJH on TLR4, I*κ*B *α*, and NF-*κ*B p65 Levels

**Figure 10:**
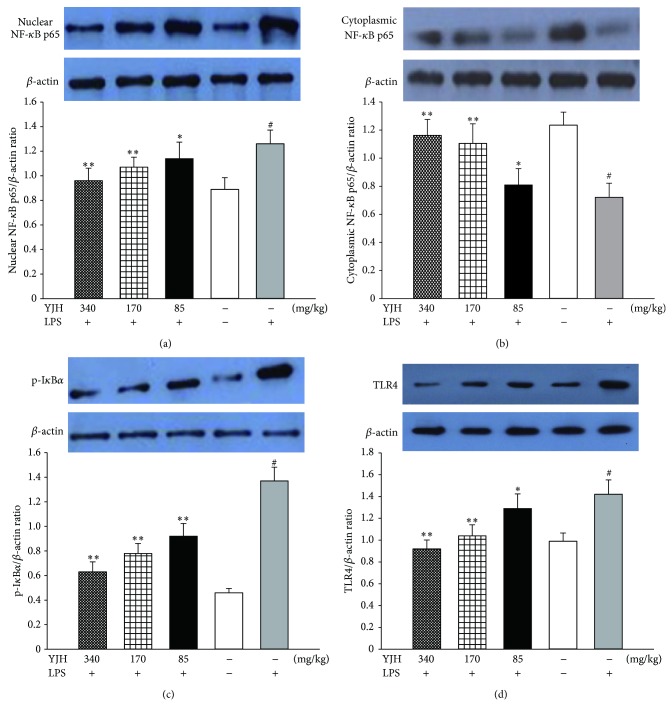
Effects of YJH on TLR4, I*κ*B *α* and NF-*κ*B p65 levels. The levels of NF-*κ*B p65 in nucleus (a), NF-*κ*B p65 in cytoplasm (b), p-I*κ*B *α* (c), and TLR4 (d). Data was represented as the mean ± SEM (*n* = 10). ^#^ *P* < 0.01 compared to the sham group, ^*^ *P* < 0.05 and ^**^ *P* < 0.01 compared to the LPS group.

In order to probe the potential mechanisms of YJH in protection of LPS-induce ALI in mice, the levels of TLR4/I*κ*B *α*/NF-*κ*B p65 in lung tissue were further investigated. As shown in Figure 10, results of western blot displayed that TLR4/MyD88/NF-*κ*B signaling pathways were activated by LPS in the LPS group (for all, *P* < 0.05, versus the sham group). However, pretreatments with YJH (85, 170, and 340 mg/kg) inhibited the phosphorylation of I*κ*B *α* and the expression of TLR4, as compared to the LPS group (for all, *P* < 0.05, versus the sham group). On the other hand, in YJH (85, 170, and 340 mg/kg) groups, the expressions of p65 subunit NF-*κ*B in lungs were downregulated in nucleus and upregulated in cytoplasm (for all, *P* < 0.01, versus the LPS-group), as shown in Figure 10. In this study, results showed that pretreatment with YJH (85, 170, and 340 mg/kg) possessed effective down-regulations of TLR4 and blocked TLR4-mediated NF-*κ*B signaling pathway efficiently, in a LPS-induced ALI mouse model.

## 4. Discussions

YJH has emerged as a drug and been applied for treatments of acute respiratory system diseases, with remarkable anti-influenza and anti-inflammation effects. In this current study, we found that pretreatments with YJH effectively prevented LPS-induced ALI changes in mice and exerted potential protective actions. Also, we successfully established the chemical analysis and chromatographic fingerprints of YJH for quality evaluation and control.

The principal findings are as follows: YJH abated LPS-induced lung histopathologic changes, declined the W/D ratio and proinflammatory cytokines productions, inhibited inflammatory cells migrations and protein leakages into the lungs, suppressed the levels of MPO, and downregulated the activations of NF-*κ*B and the expressions of TLR4.

LPS challenge leads to the leakage of serous fluid into lung tissue, resulting in typical symptoms of acute inflammation, the pulmonary edema, and is always evaluated via measuring a representative index, the lung W/D ratio in ALI mice [39]. Based on the dose-dependent attenuation of the lung wet/dry ratio in YJH (85, 170, and 340 mg/kg) pretreated groups, YJH showed a significant inhibition of the pulmonary edema in ALI mice [39]. On the other hand, as an index of lung permeability, the downregulation of BALF total protein content also demonstrated that pretreatments with YJH possessed attenuations on lung permeability enhanced by LPS [40]. To some extent, YJH inhibited the hyaline membrane formation. LPS challenge directly stimulates the infiltrations of inflammatory cells [40]. In particular, neutrophils migrating into the lung parenchyma and alveolar space have been indicated to play critical roles in the process of ALI [40, 41]. Neutrophils excrete MPO into the extracellular medium, resulting in the generation of MPO-derived oxidants and leading to lung tissues damage [40, 42]. Therefore, the infiltration of neutrophils could be represented by the MPO abilities [40, 42]. As expected, data showed that mice exposed to LPS presented massive infiltrations of inflammatory cells, including neutrophils and macrophages into the lungs. However, pretreatment with YJH significantly reversed these by decreasing the numbers of related cells and abating LPS-induced increasing of MPO activity, in the lung tissues. These results also further proved that neutrophil respiratory burst and lung tissue damage were attenuated by YJH. In addition, results of histopathologic examinations in lung tissues revealed that inflammatory responses and lung injuries in ALI mice, including pulmonary edema, alveolar distortion, hyaline membrane formation, neutrophil infiltration, hemorrhage, and necrosis, were all attenuated by YJH pretreatment, in a dose-dependent manner. In sum, results of the alveolar-capillary barrier and inflammatory response as well as histological evidences demonstrated that YJH possessed significantly protective effects on LPS-induced ALI in mice.

Clinical and experimental studies suggest that LPS induce the activation of alveolar macrophages and endothelial cells, leading to the production of several inflammatory and chemotactic cytokines in the early phases of inflammatory responses [14, 43]. TNF-*α*, IL-1*β*, and IL-6 are well-characterized cytokines involved in the process of ALI [44, 45]. These cytokines, as well as other proinflammatory compounds, further stimulate neutrophils infiltrations into lung tissues and initiate, amplify, enlarge, and perpetuate the entire or focal inflammatory responses in ALI [14, 46]. TNF-*α*, mainly produced by monocytes/macrophages, is the earliest and primary endogenous mediator of the process of an inflammatory reaction and can elicit the inflammatory cascade, cause damage to the vascular endothelial cells, and induce alveolar epithelial cells to produce other cellular factors, such as IL-6 [46, 47]. Elevated TNF-*α* binds with a TNF-*α* acceptor in lung tissue, leading to the leakage of enzymes out of the organelle, which causes damage to the lung parenchyma [45, 48]. IL-1*β* plays a key role in the progression of acute lung injury [45, 48]. It can inhibit fluid transport across the distal lung epithelium to cause surfactant abnormalities and to increase protein permeability across the alveolar-capillary barrier [45, 48]. IL-6 is one of the most common inflammatory cytokines, and its circulating levels have been shown to be excellent predictors of the severity of acute respiratory distress syndrome of different aetiologies, such as sepsis and acute pancreatitis [45, 48, 49]. In this present study, YJH significantly inhibited the productions of TNF-*α*, IL-1*β*, and IL-6 in lung. The inhibitions of proinflammatory factors productions were in accordance with the protective effects of YJH in previous analysis of inflammatory responses and histopathologic changes. And the suppression of proinflammatory cytokines of YJH was supposed to contribute to its protective effects against ALI.

NF-*κ*B is an important nuclear transcription factor and plays pivotal roles in immune and inflammatory responses through the regulation of the expression of several proteins, including proinflammatory cytokines, chemokines, and adhesion molecules [12, 45, 48, 50]. Uncontrolled activation of the NF-*κ*B pathways is involved in the pathogenesis of many acute and chronic inflammatory diseases [12, 45, 48, 50, 51]. NF-*κ*B is normally sequestered in the cytoplasm by a family of inhibitory protein known as inhibitors of NF-*κ*B (I*κ*Bs) [52]. Once activated, NF-*κ*B unit p65 dissociates from its inhibitory protein I*κ*Bs and translocates from the cytoplasm to the nucleus where they may trigger the transcription of specific target genes such as TNF-*α*, IL-1*β*, and IL-6 [6, 52]. To detect the inhibitory mechanism of TNF-*α*, IL-1*β*, and IL-6 production, we tested the effects of YJH on NF-*κ*B activations and I*κ*B *α* degradations. With the stimulation of LPS, the levels of phosphorylation of I*κ*B *α* and NF-*κ*B p65 proteins were remarkably increased. However, this tendency was reversed by YJH, supported by western blot analysis of YJH groups that I*κ*B *α* degradation and NF-*κ*B p65 activation were significantly blocked. Moreover, YJH inactivated the NF-*κ*B p65 and I*κ*B *α* phosphorylation in a dose-dependent manner. Therefore, we speculated that the protective effects of YJH against LPS-induced ALI, to some extent, may be attributed to the downregulation of NF-*κ*B pathways.

Serving as an important pattern recognition receptor of host immune responses and essential upstream sensor for LPS from pathogens and microorganisms, TLR4 would detect LPS and then trigger TLR4 signal pathways [5, 38] and via a MyD88-dependent and/or a MyD88-independent pathway, TLR4 could initiate the activation of NF-*κ*B cascades, leading to the overproductions of proinflammatory cytokines and the recruitment of inflammatory cells [5, 38, 52, 53]. Thus, TLR4 is fundamental upstream sensor for LPS, and it was valuable to probe whether YJH exerted the anti-inflammation action though TLR4-mediated pathways. Our data showed that the enhanced expression of TLR4 by LPS challenge was significantly downregulated with pretreatments of YJH at the doses of 170 and 340 mg/kg, which corresponded with the level changes of I*κ*B *α*, NF-*κ*B p65, and other proinflammatory cytokines, in ALI mice lung tissues. Therefore, we speculated that YJH could suppress TLR4/NF-*κ*B signaling pathways, leading to reductions of proinflammatory cytokines productions and attenuations of pulmonary inflammatory responses. However, explicit regulations of TLR4 signaling pathways by YJH require further studies.

In this present research, we firstly analyzed the chemical composition and established the fingerprint analysis of YJH by GC-MS and UPLC-PAD. And most of the identified compounds in YJH sample including chlorogenic acid, galuteolin, linarin, luteolin, apigenin, patchoulol alcohol, pogostone, germacrone, curdione, and furanodiene have been reported to exhibit significant inflammatory effects [18, 28, 30–33, 48]. Most importantly, our previous researches revealed that patchouli alcohol, the largest ingredient of YJH, had remarkable anti-inflammatory activity and would protect mice against the lung injury in mice of viral infection and influenza model [18, 21–23, 25–27, 33, 48]. Thus, the chemical analysis of YJH suggested that these major components are possibly responsible for the regulation of inflammatory factors against inflammation response in LPS induced ALI.

## 5. Conclusion

In this study, we explored the potentially protective effect of YJH on LPS intratracheal instillation-induced in ALI model. Furthermore, the experimental evidence demonstrated that YJH would effectively attenuate the LPS-induced ALI in mice. And protective effects of YJH were closely associated with the downregulation of TLR4/NF-*κ*B signaling pathways. These results suggested that YJH would be a potential drug for treating ALI related diseases.

## Figures and Tables

**Table 1:** Effects of YJH on TNF-*α*, IL-1*β*, and IL-6 levels.

Groups	TNF-*α* (pg/mg protein)	IL-1*β* (ng/mg protein)	IL-6(ng/mg protein)
Sham	31.97 ± 2.47	2.36 ± 0.22	5.52 ± 0.74
LPS	123.7 ± 4 9.36^#^	13. 41 ± 1.13^#^	20.20 ± 2.47^#^
Dex (5 mg/kg)	79.81 ± 13.56^**^	5.28 ± 0.61^**^	9.66 ± 1.57^**^
YJH (340 mg/kg)	82.86 ± 7.83^**^	6. 13 ± 0.53^**^	11.74 ± 1.51^**^
YJH (170 mg/kg)	91.71 ± 10.32^**^	8.14 ± 0.71^**^	13.96 ± 1.40^**^
YJH (85 mg/kg)	112.49 ± 7.53	9. 35 ± 0.94^*^	15.26 ± 1.22^**^

Data represented the mean ± SEM (*n* = 10). ^#^ *P* < 0.01 compared to the sham group; ^*^ *P* < 0.05 and ^**^ *P* < 0.01 compared to LPS group.
